# Systemic sclerosis markedly impairs health-related quality of life: a single-centre tertiary cohort study with literature-based comparison to leading causes of global disease burden

**DOI:** 10.7189/jogh.16.04228

**Published:** 2026-10-23

**Authors:** Begonya Alcacer-Pitarch, Ruben Duarte-Fernandes, Anthony C Redmond, Cristina Rocamora-Gisbert, Elizabeth Hensor, Francesco Del Galdo, Maya H Buch, Anne-Maree Keenan

**Affiliations:** 1 Rheumatology Department, Leeds Teaching Hospitals NHS Trust, Leeds, UK; 2 NIHR Leeds Biomedical Research Centre, Leeds Teaching Hospitals Trust, Leeds, UK; 3 Leeds Institute of Rheumatic and Musculoskeletal Medicine, University of Leeds, Leeds, UK; 4 Rheumatology Department, Hospital Universitari Germans Trias i Pujol, Badalona, Spain; 5 Centre for Musculoskeletal Research, Division of Musculoskeletal and Dermatological Sciences, Faculty of Biology, Medicine & Health, University of Manchester, Manchester, UK; 6 School of Healthcare, University of Leeds, Leeds, UK

**Keywords:** systemic sclerosis, quality of life, disability, SF-36

## Abstract

**Background:**

Systemic sclerosis (SSc) is a rare autoimmune condition that significantly impacts the physical and mental health of affected individuals, disrupting their daily life and overall well-being. However, its impact on health-related quality of life (HRQoL) has not been thoroughly compared to other chronic diseases, such as rheumatoid arthritis (RA) and stroke, the impact of which is clearly established. We explored how SSc influences HRQoL and compared its effect to that of six chronic diseases, considered the global leading causes of disease burden in high-income countries, and three common rheumatic and musculoskeletal diseases.

**Methods:**

We collected HRQoL data from SSc patients enrolled in a cross-sectional observational study at The Leeds Teaching Hospital NHS Trust, UK. We used the Medical Outcomes Study Short Form-36 to measure HRQoL and compared the participants’ scores to published data on unipolar major depression, ischaemic heart disease, stroke, chronic obstructive pulmonary disease, diabetes, lung cancer, osteoarthritis, RA, and lower back pain. Mean norm-based transformed scores (NBS) were compared for all eight SF-36 health domains.

**Results:**

The 121 SSc patients (106 female; mean age = 57 years, standard deviation = 12.3), disease duration mean = 9.50 years, standard deviation = 7.4) in our study reported significantly worse perceived general health (mean (x̄) NBS = 32.82) than the other nine diseases reported in the literature. Role limitations due to physical problems (x̄ NBS = 34.77) were comparable to stroke, RA, and lung cancer. Significant impairments were noted in bodily pain (x̄ NBS = 36), similar to osteoarthritis, RA, and lower back pain, as well as vitality (x̄ NBS = 39.69), comparable to RA. Emotional role limitations (x̄ NBS = 41.78) were second only to major depression, similar to stroke and RA

**Conclusions:**

We found that SSc has a substantial impact on HRQoL, resulting in significant physical and psychological impairment comparable to that experienced by patients with RA and stroke. These findings contextualise the burden of SSc and may inform future research and clinical practice aimed at improving patient care.

Systemic sclerosis (SSc) is a heterogeneous connective tissue disease characterised by vasculopathy; immune activation with production of autoantibodies and alteration in immune cells; and fibrosis of the skin and internal organs [1]. The complex and multisystem nature of the disease can have a substantial effect on both body structure and function, leading to restrictions in participation in work, social, and daily activities, as well as increased perceived disability [2,3]. However, for patients with SSc, perceived disability does not always reflect disease severity, as clinical factors and psychological impact are not linearly related [2,3]. This indicates that the patients’ perception of the impact of the disease manifests as a complex interaction between the physical, psychological, and psychosocial factors, as well as personality traits and individual disease perception [4,5].

In the context of SSc, this complexity is evident in the diverse factors associated with disability. Some clinical features correlate predominantly with physical disability, others with psychological disability, and some influence both, although findings vary across studies [5–7]. Physical disability has been associated with joint contractures, digital ulcers, extremity ulcers (other than digital), dyspnoea, gastrointestinal involvement, joint pain on motion, joint tenderness, tender point count, and high skin score [6–9]. Several of these symptoms also relate to impaired psychological function. For example, skin involvement seems to be significantly associated with dissatisfaction with appearance and social discomfort [10]. Digital ulcers may also contribute to aesthetic concerns [11], not only because of their visible appearance but also because scarring can result in permanent tissue loss, nail abnormalities, and digital deformities, which may further affect body image and social interactions. Similarly, fear of negative evaluation , defined as concern about others' negative judgments and reactions, particularly regarding disease-related changes in appearance or function [12], and lung involvement has been linked to anxiety, while overall SSc severity has been linked to depression [13]. Depression and anxiety have further been associated with skin itchiness [14], as well as pain, fatigue, reduced social support, helplessness, fear of disease progression, and perceived attractiveness [13,15]. Furthermore, although clinical severity does not directly predict psychological impact [2,3,8,13], functional disability has been identified as a significant predictor of distress in patients with SSc [9,16], reinforcing the importance of disability as a key determinant of psychological well-being.

Together, this physical and psychological burden contributes to a broader psychosocial impact that extends beyond traditional clinical outcomes. This impact is commonly referred to as a patients’ health-related quality of life (HRQoL), a multi-dimensional concept encompassing the individual’s perceptions of physical, mental, emotional and social functioning, as well as levels of independence. The concept of HRQoL captures how health status affects overall quality of life (QoL) beyond the direct measures of morbidity, mortality, or life expectancy [17].

Existing literature indicates that SSc negatively affects patients’ HRQoL [18,19], with reduced physical function as a significant contributor to its deterioration [20]. Factors such as joint involvement, >11 tender points, gastrointestinal involvement, and high skin scores (indicating worse skin fibrosis) have been found to adversely affect physical function and HRQoL [7]. Furthermore, Raynaud’s disease, gastrointestinal symptoms, and digital ulcers as independent risk factors for worsening of HRQoL over time [20]. Finally, compared to individuals with other rheumatic diseases, patients with SSc report greater impairment in mental health and poorer general health perception [21].

Besides evidence showing the detrimental impact of the disease at a physical and psychological level, and its effect on patients’ HRQoL, the health needs of patients with SSc continue to be unmet [22–24]. Therefore, evaluating HRQoL is particularly important, as it reflects aspects of disease burden that may not be evident through clinical assessment alone and provides insight into domains of functioning that matter directly to patients [17].

In this study, we aimed to evaluate HRQoL, as assessed by the Medical Outcomes Study (MOS) Short Form-36 (SF-36) patient-reported outcome measure, in a cohort of patients with SSc, and to compare their scores with SF-36 data extracted from previously published studies of nine chronic diseases, including the six leading causes of the global disease burden in high-income countries and three common rheumatic and musculoskeletal diseases (RMDs).

## METHODS

### Participants

 We collected cross-sectional data between February 2011 and 2013 from 121 patients diagnosed with SSc as per 2013 EULAR/ACR classification criteria [25], who were attending a connective tissue disease outpatient clinic at Leeds Teaching Hospital NHS Trust, UK. Patients were consecutively enrolled into observational study and asked to complete the MOS SF-36, version 1, on the same day, to obtain their HRQoL data. The MOS SF-36 is a validated generic health questionnaire designed to assess a person’s perceived general health status. It consists of eight health domains which score three aspects of health: functional status, which comprises physical functioning, role limitations due to physical health problems, social functioning, and role limitations due to emotional problems; well-being, encompassing general mental health (psychological distress and psychological well-being), bodily pain, and vitality (energy/fatigue); and the overall evaluation of health, captured by a general health perception domain [26–28]. Scores obtained from the questionnaire range from 0, representing the worst health state measurable, to 100, indicating the best health state. As the SF-36 contains generic patient-reported outcomes, it allows for its scores to be compared across different diseases [27–29]. The scores obtained were compared to those from published studies describing the HRQoL of nine chronic diseases. Of these, six were considered among the global leading causes of burden of disease in high-income countries [30]: unipolar major depression (MD), ischaemic heart disease (IHD), stroke, chronic obstructive pulmonary disease (COPD), diabetes, and lung cancer (LC). The three remaining diseases were major RMDs: osteoarthritis (OA), rheumatoid arthritis (RA), and lower back pain (LBP).

### Criteria for study selection

To conduct the comparison, we identified studies by searching Embase, MEDLINE (*via* Ovid), and PubMed using terms related to the MOS SF-36, QOL, HRQoL, and the name for each of the nine identified diseases (Figure S1 in the **Online Supplementary Document**). We only included studies written in English where the population was ≥19 years old, the research was conducted in the UK, and the data from all domains of the SF-36 were reported using the mean as a measure of central tendency. To avoid differences in health states associated with nationwide changes in healthcare delivery over time, we selected studies in which data collection occurred within 15 years of our research [31-39]. This was achieved in all but the COPD study [31], which reported scores from 18 years before the current data (**Table 1**).

**Table 1:** Summary of the characteristics of the ten comparative studies

Disease studied	Population	Sample size	Age in years, x̄	Period of data collection	Study design	Numberof women (%)	Sample disease duration/characteristics
SSc	UK	121	57.13	2011–2013	Cross-sectional observational	106 (87.6)	x̄ (SD) = 9.50 (7.4) years
OA Knee & Hip [32]	UK	763	69.5	2002–2003	Multicentre, cross-sectional observational	433 (56.7)	Not specified
RA [33]	UK	446	59.58	2002–2008	Multicentre, cross-sectional observational	336 (75.0)	Early and Established RA
Lower back pain [34]	UK	144	41.7	1997–2001	Parallel-group, pragmatic multi-centre RCT	83 (57.6)	Lower back pain for at least six weeks
Ischaemic heart disease [35]	North of Ireland (Great Britain)	117	60.61	2003	Cross-sectional observational	45 (38.6)	Mdn (range) = 6 months (2 days to 42 years)
Stroke [36]	UK	217	69.5	1997–1999	Multicentre, parallel group, double-blind RCT	128 (48.5)	Acute ischaemic stroke; time to randomisation in hours: x̄ (SD) = 30.6 (12.5)
COPD [31]	UK	376	64	1992–1995	Multicentre, parallel-group double-blind RCT	185 (32.9)	Moderate to severe chronic obstructive pulmonary disease
Major depression (unipolar) [37]	UK	250	37.4	1999	Multicentre, parallel-group RCT	190 (76.0)	Moderate level of depression
Diabetes [38]	UK	799	Reported as <45 to ≥75	Not reported	Cross-sectional questionnaire survey	390 (48.81)	Type 2 diabetes: 0 to ^3^ 20 years
Lung cancer [39]	UK	45	62.4	1999	Cross-sectional, multi-centre study	13 (30.0)	x̄ (SD) months = 4.4 (4.0)

Mdn – medianOPD – chronic obstructive pulmonary disease, OA – osteoarthritis, RA – rheumatoid arthritis, RCT – randomised controlled trial, SD – standard deviation, SSc – systemic sclerosis, x̄ – mean

### SSc data collection and statistical analysis

A clinical research fellow (RW) collected medical data from patients with SSc at the time of assessment, which were subsequently verified through review of medical records. The SF-36 questionnaires were scored using QualityMetric Health Outcomes^TM^ Scoring Software 4.0 (QualityMetric Incorporated, Lincoln, Rhode Island, USA), which applies validated SF-36 scoring algorithms to calculate domain scores and summary physical and mental health component scores. Mean scores for the eight SF-36 domains were transformed into norm-based scores (NBSs) using general US population norms [40]. We used normalisation against the US population because Ware *et al*. [41] demonstrated that there is a substantial relative agreement between SF-36 summary measures estimated using standard (US derived scoring algorithms) and country-specific (UK) scoring algorithms, enabling comparison of average scores across countries. After transformation, an NBS of 50 represents the mean of the general population (standard deviation (SD) = 10), while scores more than 10 points above or below 50 indicate substantially better or worse health status, respectively.

We used Student’s independent groups *t*-tests, adjusted for unequal group sizes, to compare each condition to SSc, without formal adjustment for multiple comparisons. We considered differences of ≥5 units (equivalent to 0.5 SD in the US general population) to be clinically meaningful. For all of the conditions except LC, differences of ≥5 units were statistically significant at *P* < 0.001; for LC, with a smaller sample size and lower power, differences of ≥5 units were statistically significant at *P* < 0.05, and differences of ≥10 units were statistically significant at *P* < 0.001. We performed all analyses in Stata, version 18.0 (StataCorp LLC, 2023. College Station, Texas, USA).

## RESULTS

For the SSc cohort, 138 patients were approached to participate in the study, of whom 16 declined to participate due to ill health or not having personal capacity, while 1 was withdrawn from the study because of misdiagnosis. We included 121 participants with SSc (106 female; median age = 59 years, interquartile range (IQR) = 25–86) with a median disease duration of 9 years (IQR = 4–13) and a median modified Rodnan Skin Score of 2 (IQR = 0–51). In terms of specific disease subtypes, 96 (79%) patients had limited cutaneous SSc, 24 (20%) had diffuse cutaneous SSc, and 1 (0.8%) had SSc *sine* *scleroderma* (**Table 2**).

**Table 2:** Demographic characteristics of patients with SSc (n = 121)*

	Value
**Age in years, x̄ (SD)**	57 (12.3)
**Male/female**	15(12.4)/106 (87.6)
**Ethnicity**	
White	109 (89.7)
Indian	6 (5)
Pakistani	5 (4)
Other ethnic group	1 (0.8)
**BMI in kg/m^2^, x̄ (SD)**	26.29 (5.6)
**Smoking history**	56(47.9)
**Number of comorbidities, x̄ (SD)**	4.65 (2.4)
**Disease duration in years, x̄ (SD)**	9.50 (7.4)
**Disease subtype**	
Limited SSc	96 (79.0)
Diffuse SSc	24 (20.0)
SSc *sine* *scleroderma*	1 (0.8)
**mRSS, Mdn (IQR)**	2 (0–51)
**Patients with a history of skin ulcers**	59 (49.0)
**Patients with current ulcers**	18 (15.0)
**Patients with a history of calcium deposits**	57 (48.0)
**Patients with current calcium deposits**	39 (32.0)
**Medications**	
Immunosuppressant	29 (24.0)
Methotrexate	26 (21.0)
Mycophenolate Mofetil	3 (2.5)
Steroids	26 (21.0)
Calcium channel blockers	65 (54.0)
ACE inhibitors	48 (40.0)
Beta-Blockers	4 (3.0)
Bosentan	3 (2.5.0)
Sildenafil	6 (5.0)
Iloprost (within three months prior to the study)	22 (18.0)
Other medications – unclassified	5.47 (3.5)

BMI – body mass index, IQR – interquartile range, Mdn – median, mRSS – Modified Rodnan Skin Score, SD – standard deviation

*Presented as n (%) unless specified otherwise.

The most common SSc associated manifestations were Raynaud’s phenomenon (98.4% of patients), followed by oesophageal reflux (66.1%), soft tissue calcification (47.1%), and lung fibrosis (18.2%). The most common co-existing conditions included hypertension (19.8%), OA (11.6%), depression (9.1%), history of previous cancer (5.8%), diabetes (4.1%), COPD (4.1%), lower back pain (2.5%) RA (0.8%), and IHD (0.8%) (**Table 3**).

**Table 3:** Comorbidities for patients with SSc (n = 121) per ICD-11 classification (WHO)

Diseases of circulatory system	Value
Raynaud phenomenon	119 (98.4)
Hypertension, arterial	24 (19.8)
Heart murmur	4 (3.3)
Tachycardia	1 (0.8)
Ischaemic heart disease	1 (0.8)
Cardiac effusion	1 (0.8)
Angina	1(0.8)
**Diseases of the digestive system**	
Reflux	80 (66.1)
Bowel involvement	10 (8.3)
Hiatus hernia	7 (5.8)
Oesophageal dysmotility	5 (4.1)
Barrett’s oesophagus	5 (4.1)
Oesophagitis	2 (1.7)
Liver cirrhosis	2 (1.7)
Gave stomach	1 (0.8)
Gastritis	1 (0.8)
Diverticulitis	1 (0.8)
Crohn disease	1 (0.8)
Constipation	1 (0.8)
Soft tissue calcifications	57 (47.1)
Psoriasis	3 (2.5)
Eczema	3 (2.5)
Lichen sclerosus	1 (0.8)
Lung fibrosis	22 (18.2)
Pulmonary hypertension	6 (5.0)
COPD	5 (4.1)
Asthma	5 (4.1)
Interstitial lung disease	4 (3.3)
Emphysema	3 (2.5)
Tuberculosis	1 (0.8)
Sarcoidosis	1 (0.8)
Pulmonary embolism	1 (0.8)
Bronchiectasis	1 (0.8)
Hay Fever	1 (0.8)
**Diseases of the musculoskeletal system or connective tissue**	
Osteoarthritis	14 (11.6)
Osteoporosis	10 (8.3)
Arthritis	6 (5.0)
Fibromyalgia	5 (4.1)
Back pain	3 (2.5)
Hypermobility	2 (1.7)
Gout	2 (1.7)
Rheumatoid arthritis	1 (0.8)
Osteopenia	1 (0.8)
Still disease	1 (0.8)
Polyarthralgia	1 (0.8)
Scoliosis	1 (0.8)
Costochondritis	1 (0.8)
Degenerative spine	1 (0.8)
**Endocrine, nutritional, or metabolic disorders**	
Hypothyroidism	17 (14.1)
Hyperlipidaemia	6 (5.0)
Diabetes	5 (4.1)
Vitamin D deficiency	1 (0.8)
**Diseases of the immune system**	
Sjogren’s	13 (10.7)
Systemic lupus erythematosus	3 (2.5)
Dermatomyositis	2 (1.7)
Polymyositis	1 (0.8)
Vasculitis	1 (0.8)
**Mental, behavioural, or neurodevelopmental disorders**	
Depression	11 (9.1)
Anxiety, generalised	5 (4.1)
**Nervous system diseases**	
Migraine	3 (2.5)
Peripheral neuropathy	2 (1.7)
Vertigo	1 (0.8)
Trigeminal neuralgia	1 (0.8)
Epilepsy	1 (0.8)
Amaurosis fugax	1 (0.8)
Multiple sclerosis	1 (0.8)
**Neoplasms**	
Cancer History	7 (5.8)
**Diseases of the blood or blood forming organs**	
Anaemia	6 (5.0)
**Diseases of the genitourinary system**	
Kidney failure	1 (0.8)
Renal disease	1 (0.8)
**Symptoms, signs, or clinical findings not elsewhere classified**	
Pain, chronic	1 (0.8)
Chronic fatigue	1 (0.8)
**Sleep-wake disorders**	
Sleep apnoea	1 (0.8)

COPD – chronic obstructive pulmonary disease, SSc – systemic sclerosis, WHO – World Health Organization

### Functional status

For physical function, stroke, LC, RA and SSc patients scored the lowest, with mean NBSs of 27.23, 27.78, 28.27, and 30.78, respectively, meaning they had the poorest physical function (**Figure 1**). We found no statistically significant differences between these four diseases (NBS difference <5 units), indicating that they all experiencing similarly poor physical health. We also found no statistically significant difference between SSc, COPD, and OA, with mean NBSs of 30.78, 32.30 and 34.92.

**Figure 1:**
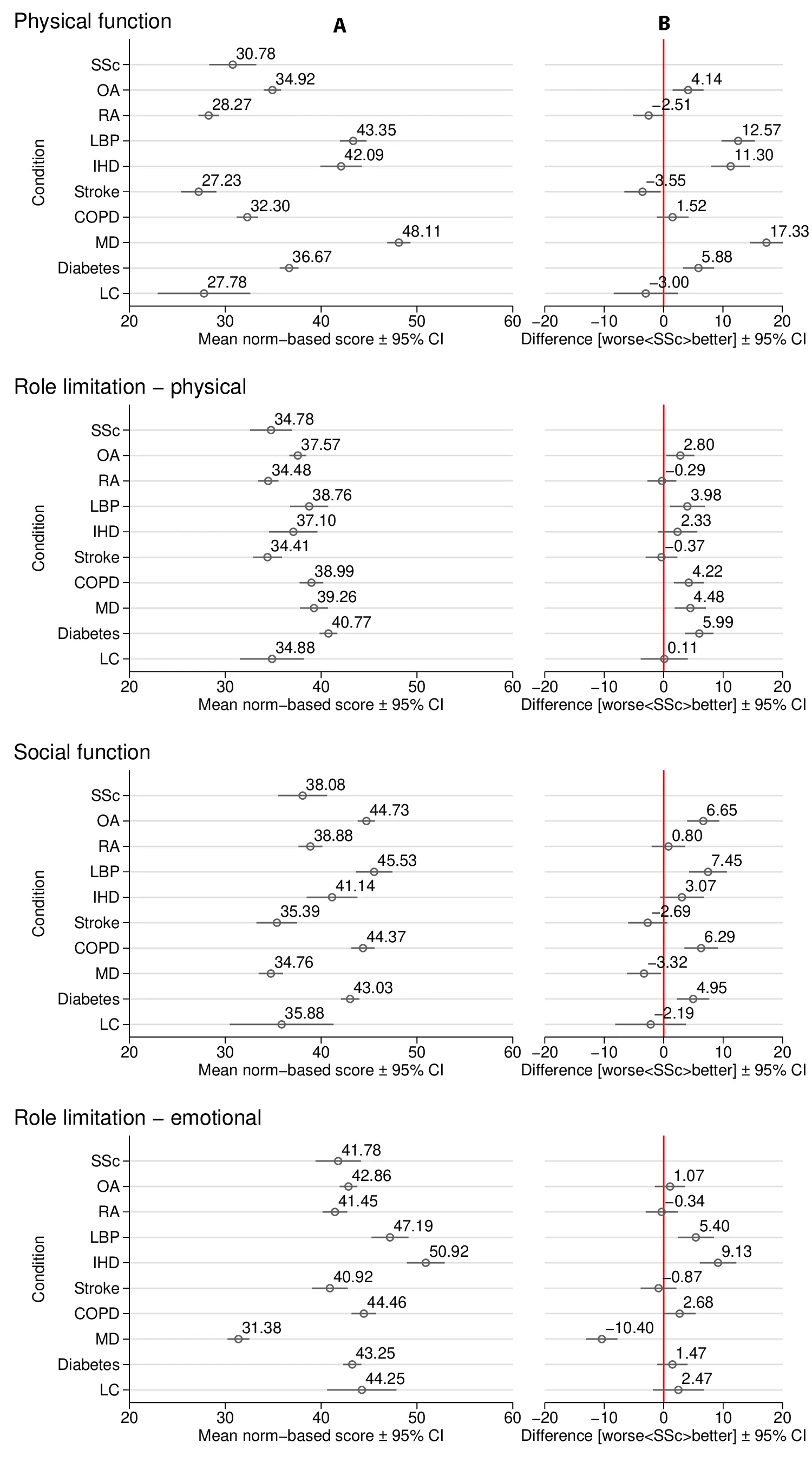
Health domains measuring functional status. Error bar plots of mean (95% CI) norm-based scores for the 10 conditions for each of the four health domains measuring function status (**Panel A**) and differences between each condition and SSc (**Panel B**). Positive differences represent better health-related quality of life compared to SSc. CI – confidence interval, COPD – chronic obstructive pulmonary disease, IHD – ischaemic heart disease, LBP – lower back pain, LC – lung cancer, MD – major depression, OA – osteoarthritis, RA – rheumatoid arthritis, SSc – systemic sclerosis.

SSc patients showed significantly worse physical function (NBS difference >5 units) than MD (mean (x̄) NBS) = 48.11, NBS difference = +17.33 units), LBP (x̄ NBS = 43.35, NBS difference = +12.57), IHD (x̄ NBS = 42.09, NBS difference = +11.30), and diabetes (x̄ NBS = 36.67, NBS difference = +5.88). For MD, LBP, and IHD, the participants scored within one SD of the general population, thus experiencing a physical function similar to that of the general population.

We noted similar results in the domain for role limitation because of physical problems. Stroke patients reported the lowest scores (x̄ NBS = 34.41), together with RA (x̄ NBS  = 34.48), SSc (x̄ NBS = 34.78), and LC patients (x̄ NBS = 34.88), with no statistically significant difference. The IHD and OA patients had mean NBSs of 37.10 and 37.57, respectively. The rest of the diseases – LBP, COPD, MD, and diabetes – had higher mean scores of 38.76, 38.99, 39.26, and 40.77, respectively, indicating lesser impairment than among SSc patients in role limitation because of physical function. However, only the difference for diabetes was statistically significant (NBS difference = +5.99 units).

For the social functioning health domain, patients with unipolar MD, stroke, and LC had the lowest mean NBS scores of 34.76, 35.39, and 35.88, with SSc and RA patients also reporting impaired social functioning at mean NBSs of 38.08 and 38.88, respectively. However, we found no statistically significant differences in NBS between groups. Patients with IHD (x̄ NBS = 41.14), diabetes (x̄ NBS = 43.03), COPD (x̄ NBS = 44.37), OA (x̄ NBS = 44.73) and LBP (x̄ NBS = 45.53) scored higher on the social functioning domain. There was a significant difference for COPD, OA, and LB when compared to SSc patients, with NBS differences of +7.45, +6.65, and +6.29, respectively. We found no statistically significant difference for diabetes.

When considering role limitations due to emotional problems, patients with unipolar MD reported more limitations (x̄ NBS = 31.38), with significantly worse NBSs than those reported by SSc patients (NBS difference = −10.40). Stroke, RA, and SSc patients had very similar mean NBSs of 40.92, 41.45, and 41.78, respectively, with no significant difference present between them. Patients with SSc showed worse scores than those with OA (x̄ NBS = 42.86), diabetes (x̄ NBS = 44.25), LC (x̄ NBS = 44.25), and COPD (x̄ NBS = 44.46), while patients with LBP (x̄ NBS = 47.19) and IHD (x̄ NBS = 50.92) reported the least impairment of all groups in role limitations associated with emotional problems.

Although most of the disease groups tended to have better outcomes in this domain than SSc patients, SSc scores were only statistically significantly worse than those from LBP patients (NBS difference = +5.40) and IHD (NBS difference = +9.13). Except for the MD group, all scores were within or close to one SD of the general population, indicating their role limitations due to emotional problems were similar to those experienced by the general population.

### Well-being

Well-being is encompassed by three health domains: general mental health, bodily pain, and vitality health (**Figure 2**). Apart from MD (x̄ NBS = 27.27), the other diseases scored within one SD of the general population. Patients with SSc demonstrated the second worst general mental health score (x̄ NBS = 44.54), similar to patients with RA (x̄ NBS = 44.53). Other conditions reporting lower scores than the general population were stroke (x̄ NBS = 45.09), diabetes (x̄ NBS = 46.20), LBP (x̄ NBS = 47.31), IHD (x̄ NBS = 47.62), COPD (x̄ NBS = 48.98), OA (x̄ NBS = 49.48), and LC (x̄ NBS = 50.08). We only noted significant differences between SSc and LC (NBS difference = +5.55 units). We found no statistically significant difference for OA. Only patients with MD had statistically worse results (NBS difference = −17.27) than SSc patients.

**Figure 2:**
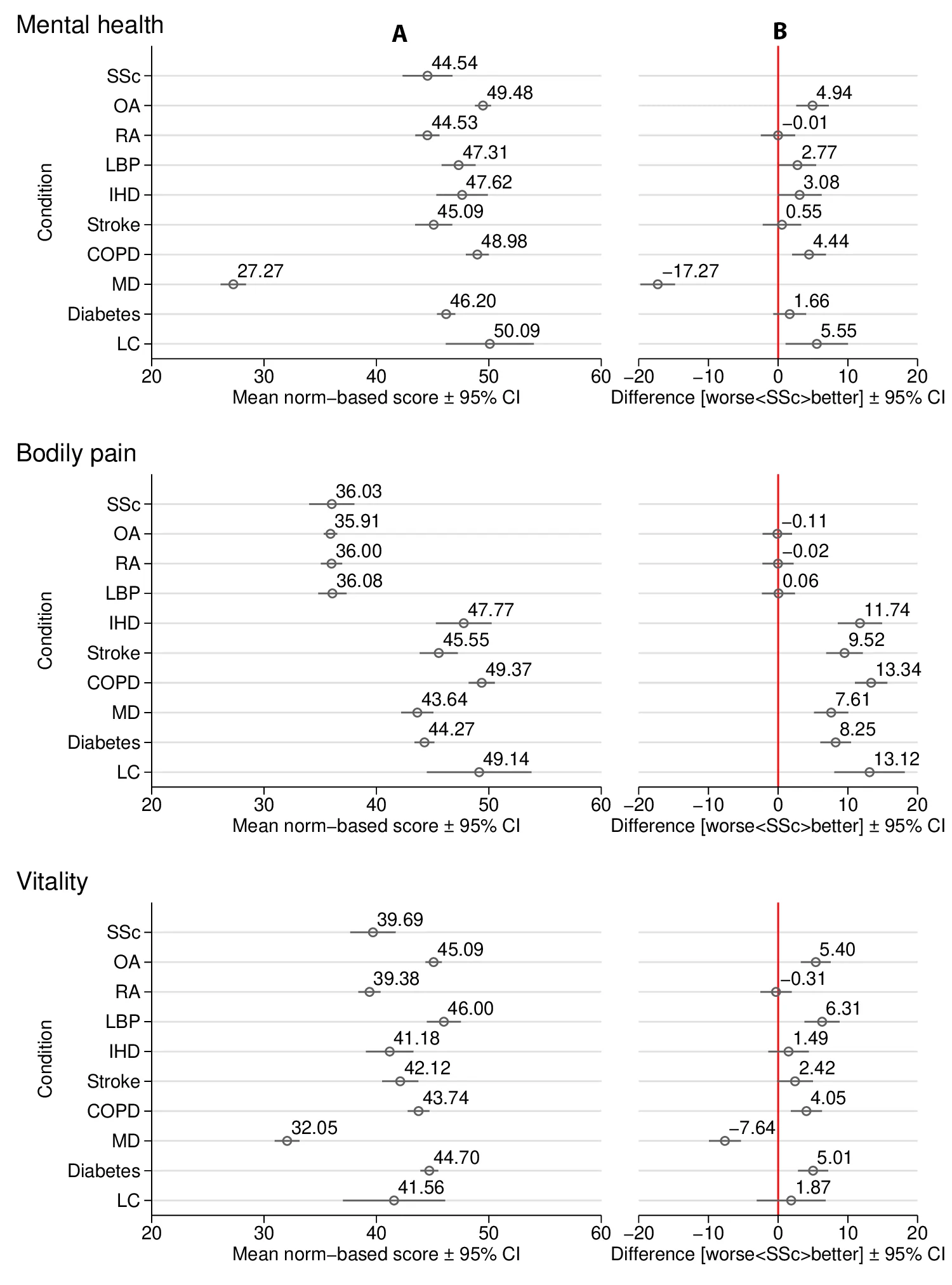
Health domains measuring well-being status. Error bar plots of mean (95% CI) norm-based scores for the 10 conditions for each of the three health domains measuring well-being status (**Panel A**) and differences between each condition and SSc (**Panel B**). Positive differences represent better health-related quality of life compared to SSc. CI – confidence interval, COPD – chronic obstructive pulmonary disease, IHD – ischaemic heart disease, LBP – lower back pain, LC – lung cancer, MD – major depression, OA – osteoarthritis, RA – rheumatoid arthritis, SSc – systemic sclerosis.

For the bodily pain domain, the lowest scores were associated with the RMDs, with all four conditions – OA (x̄ NBS = 35.91), RA (x̄ NBS = 36), SSc (x̄ NBS = 36.03), and LBP (x̄ NBS = 36.08) reporting mean NBSs around 36, meaning there were no statistically significant differences between groups. The mean NBSs for the other six diseases ranged from 43 to 49, meaning they were within an SD of the general population. When compared their NBS means to SSc scores, all were statistically significantly better than SSc: the mean NBS score for MD was the highest (x̄ NBS = 43.64, NBS difference = +7.61) followed by diabetes (x̄ NBS = 44.27, NBS difference = +8.25), stroke (x̄ NBS = 45.55, NBS difference = +9.52), IHD (x̄ NBS = 47.77, NBS difference = +11.74), LC (x̄ NBS = 49.14, NBS difference = +13.12) and COPD (x̄ NBS = 49.37, NBS difference = +13.34).

Patient groups who reported the lowest vitality were those suffering from MD (x̄ NBS = 32.05), followed by those with RA (x̄ NBS = 39.38) and SSc (x̄ NBS = 39.69), for which there was no statistically significant differences NBSs. Intermediate scores falling within the scores from the general population were seen in patients with IHD (x̄ NBS = 41.18), LC (x̄ NBS = 41.56), stroke (x̄ NBS = 42.12). and COPD (x̄ NBS = 43.74). Higher scores were reported by patients with diabetes (x̄ NBS = 44.70), OA (x̄ NBS = 45.09), and LBP (x̄ NBS = 46). Furthermore, SSc patients had significantly lower vitality than LBP (NBS difference = +6.31), OA (NBS difference = +5.40), and diabetes (NBS difference = +5.01). Only patients with MD had significantly worse vitality scores than SSc patients (NBS difference = −7.64).

### Overall health

Patients with SSc perceived their health to be poorer (x̄ NBS = 32.82) than that reported by any of the other nine diseases (**Figure 3**). Patients with COPD (x̄ NBS = 35.02) and RA (x̄ NBS = 35.29) also had low scores, while the scores observed for people with diabetes (x̄ NBS = 39.98), LC (x̄ NBS = 39.99), MD (x̄ NBS = 40.92), stroke (x̄ NBS = 42.26), OA (x̄ NBS = 43.75), LBP (x̄ NBS = 47.22), and IHD (x̄ NBS = 47.345) were all within the SD of the general population. When compared to SSc group, all diseases showed statistically significant differences, except RA (NBS difference = +2.47) and COPD (NBS difference = +2.20).

**Figure 3:**
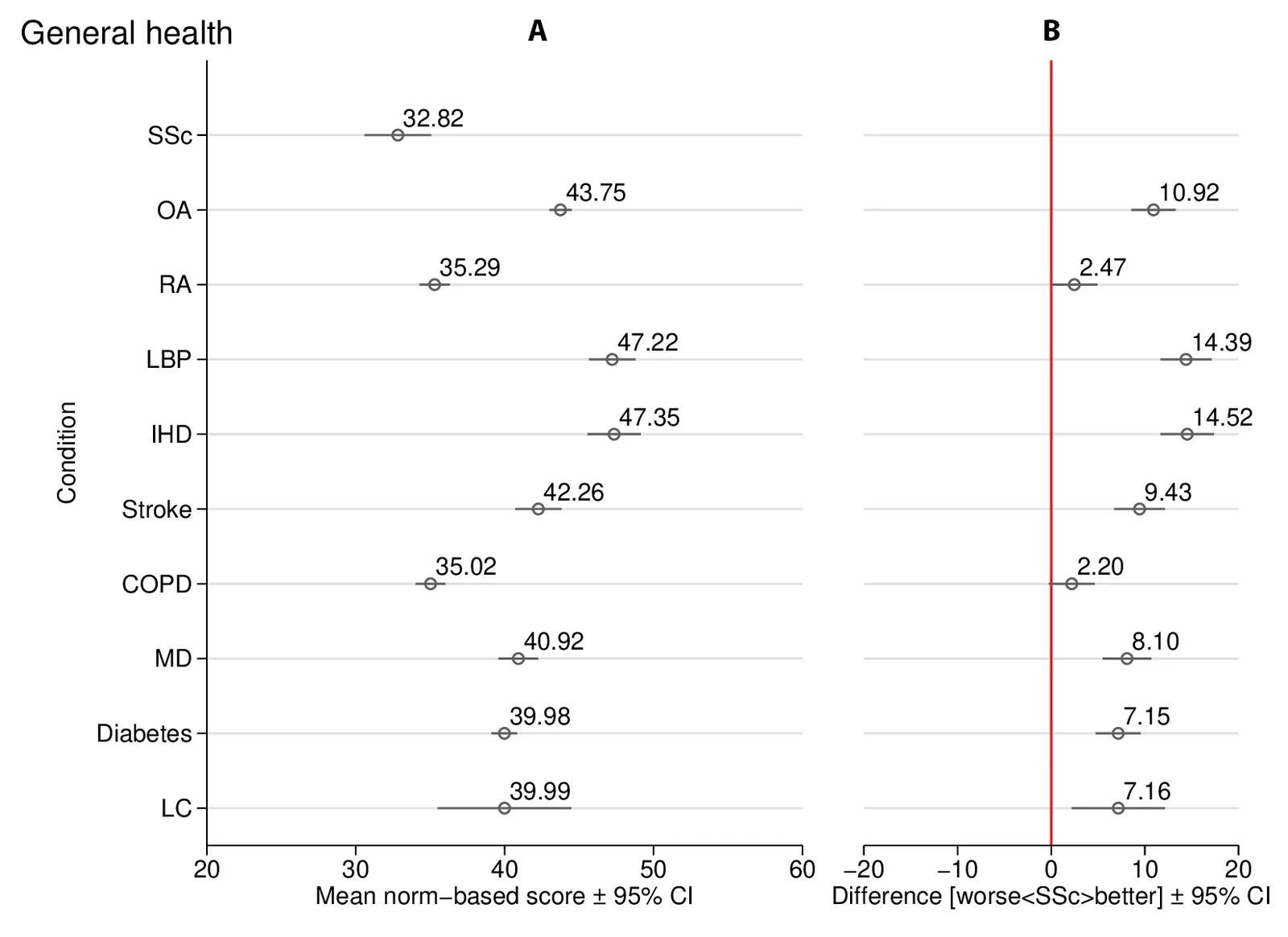
Graphic representation of the general health perception measuring overall evaluation of health. Error bar plots of mean (95% CI) norm-based scores for the 10 conditions for the general health perception domain (**Panel A**) and differences between each condition and SSc (**Panel B**). Positive differences represent better health-related quality of life compared to SSc. CI – confidence interval, COPD – chronic obstructive pulmonary disease, IHD – ischaemic heart disease, LBP – lower back pain, LC – lung cancer, MD – major depression, OA – osteoarthritis, RA – rheumatoid arthritis, SSc – systemic sclerosis.

## DISCUSSION

We sought to compare the impact of SSc on HRQoL with literature-based scores from the six chronic diseases considered among the global leading causes of burden of disease in high-income countries, as well as three major RMDs. Our results demonstrated that, while each of the ten chronic diseases investigated in this study has a substantial impact, SSc is consistently amongst the four diseases with the poorest HRQoL, and patients with SSc report the worst overall health perception.

The poor HRQoL in patients with SSc is well documented [42] and has been compared to that experienced by individuals with other rheumatic diseases such as RA, systemic lupus erythematosus, and Sjogren’s syndrome [21]. To our knowledge, no comparison has been made with the chronic disease considered among the leading causes of the global burden of disease in high-income countries.

In terms of physical impairment, it is well documented that patients with SSc suffer substantial impact on physical functioning, reflecting the multi-system nature of the disease [6–9,11]. Physical disability has been found to correlate with joint pain, joint contractures, digital and other skin ulcerations, skin fibrosis, dyspnoea, and gastrointestinal involvement [6–9]. Gastrointestinal problems have been reported as one of the major complications impacting QoL in patients with SSc and has been associated with the most negative perception of illness severity [42]. Patients with SSc often report difficulties with daily activities such as bathing, dressing, or walking. Physical functioning may also become more difficult as the disease progresses: flexion contractures and fixation of interphalangeal joints may often lead to loss of hand function and inability to undertake basic grooming activities [43,44]. Function can also be impaired by the presence of calcium deposits, which can limit joint function [44]. Lower limb involvement has been found to impact on gait parameters such as walking speed and stride length [45], which may also be compounded by cardiopulmonary involvement making walking difficult [46].

The physical limitations experienced by the patients with SSc in our study are similar to those felt by patients with stroke, LC, and RA, who reported to have the worst physical function among the reviewed diseases. While specialist multidisciplinary health services have been developed in the UK to meet the health needs of patients with stroke [47], cancer [48] and RA [49], the provision of specialist services for patients with SSc still under development [50].

The impact of physical functioning on psychological well-being is extremely important in all chronic diseases. In patients with SSc, physical function may also be affected by experienced increase pain and reduced energy. Indeed, pain has previously been reported to affect physical functioning and social adjustment in SSc – two relevant factors contributing to HRQoL [51]. In this study, the bodily pain reported by SSc patients was as high as that reported by the patients suffering from the three RMDs and significantly greater than that reported by individuals with any of the six leading causes of global disease burden in high-income countries. Fatigue represents an additional and often overlooked burden in SSc. Beyond its direct impact on quality of life, it can substantially impair participation in daily activities, including parenting, household responsibilities, and employment, and has been linked to pain, sleep disturbances, and depression [52]. Together, these interconnected symptoms may contribute to the considerable psychosocial burden associated with SSc. Consistent with this interpretation, the patients with SSc in our study reported poorer well-being than those with LC and COPD, two well-recognised diseases with large psychological impact [53–55].

On a psychological level, patients with SSc reported general mental health outcomes comparable to those of patients with RA, and poorer than those observed in the other non-psychiatric conditions. This impact was particularly evident in measures assessing limitations due to emotional problems. In this domain, patients with SSc were among the non-psychiatric disease groups experiencing the greatest limitations, but with no statistically significant differences compared to patients with stroke or RA. However, the general mental health scores for all non-psychiatric diseases, including SSc, were within one SD of the general population. These findings differ slightly from those reported in a SSc inception cohort which reported patients with SSc having worse mental health than the general population [56], but are similar to those published from a Canadian cohort of patients with SSc, for whom the mental health was comparable to that in other chronic diseases and norms [57].

These results, however, might also show how the mental health items of the SF-36, a generic questionnaire, might fail to capture SSc-specific emotional or cognitive burdens (*e.g.* distress from gastrointestinal problems, digital ulcers, Raynaud’s phenomenon, or concerns about disfigurement). This has previously been reported in the literature, where disease-specific instruments such as HADS or SSc-specific patient-reported outcomes measures have identified significant psychological distress that is often not reflected in SF-36 scores [58]. Therefore, future studies should consider the use of the SSc QoL [59] which may be better suited to capturing the broader impact of SSc on patients' quality of life. Unlike measures that are informed primarily by clinicians' perspectives, the SSc QoL was developed directly from patients' experiences and reflects issues that individuals with SSc themselves consider important, independent of physicians' interpretation of quality of life [59,60]. The instrument is a needs-based QoL measure specifically developed for people with SSc [59], meaning it proposes that QoL is determined by an individual's ability to fulfil their needs and achieve aspects of life that are important to them [61]. As such, the SSc QoL may provide a more patient-centred assessment of disease burden and better capture aspects of living with SSc that might otherwise be overlooked by generic health status measures.

Overall, patients with SSc perceived their health poorer than any of the other nine diseases groups. This may be explained by the fact that, in SSc, perceptions of health are influenced less by objective disease severity and more by patients’ beliefs and emotional responses to their condition [4]. Given this context, psychological and counselling services represent an essential component of comprehensive SSc care. Many patients face challenges related to physical disability, disfigurement, altered self-perception, and concerns about disease progression [62,63]. Access to psychological support can help individuals develop evidence-based coping strategies that maintain or improve psychological well-being [62].

The confounding interaction between physical and psychological impact has been well documented in the context of chronic diseases [64], particularly in terms of the patient’s participation in certain life-roles and or valued activities [65,66]. Our results indicate that the inability to participate is as high as that experienced by other individuals with chronic disabling diseases. Social functioning was worse in patients with SSc than the general population, as impaired as that experienced by RA patients, and not statistically different from that experienced by MD, stroke, and LC patients. Although we found no statistically significant difference in social functioning between patients with SSc and RA, published data have indicated that SSc has a worse impact on work disability than RA [67]

The inability to undertake valued activities because of physical and emotional impairment is also likely to impact social participation and increase social isolation, which in turn may lead to further mental and physical impairment [68,69]. For example, in cardiovascular disease, social isolation has been reported to increase the risk of mortality [70]. In SSc patients, it can be further hampered by external factors, such as healthcare provision arrangements. For example, attendance at hospitals to receive periodic treatments such as iloprost infusions, which varies from an eight-hour infusion to continuous infusions over three days, has previously been reported to interfere with social functioning [71]. In order to minimise the impact caused by the way health-services are being provided and meet patient health needs, it is recommended that healthcare systems adopt a patient-centred service model [72] with one-stop multi-disciplinary specialist clinics ideally embedded within an integrated care framework designed to improve health outcomes and optimise resource use [73]. These clinic models are already in use for chronic conditions such as COPD [74] and stroke [75], offering an effective way to streamline care enabling coordinated, same-day assessments by multiple relevant specialists, thereby reducing patient burden while enhancing clinical efficiency. By capturing the impact of SSc on the patients’ HRQoL and comparing it to these nine diseases, we sought to contextualise the impact of SSc and highlight the need to support patients suffering from this rare disease.

### Strengths and limitations

Some limitations must be considered when interpreting these data. There is a time gap of up to 18 years between the data collection periods of some comparator studies. Advances in healthcare over this time interval may have influenced both patient management and their perception of QoL, particularly in RA. We believe that the similar degree of HRQoL impairment observed between SSc and RA may partly reflect the period in which the RA cohort was recruited (years 2002–2008). At this time, therapeutic options to modify disease activity were more limited and the majority of the RA patients’ cohort [35] had established RA, with probable structural damage. This is relevant because several therapies such as IL-6 inhibitors, rituximab, and abatacept were only approved by the Food and Drug Administration in 2005. While immunosuppressive agents may be used off-label, comparable therapeutic advances have not been achieved in SSc, which could influence HRQoL outcomes.

Additionally, the comparator studies were not originally designed for cross-study comparisons; therefore, participant characteristics such as demographic profile, disease duration, and other clinical variables were not matched. Furthermore, we could not conduct the analysis due to the small number of patients with diffuse cutaneous SSc and *sine scleroderma* in our sample. Nonetheless, 79% of our cohort had limited cutaneous SSc, consistent with SSc cohorts in previous literature [76]. This phenotype is generally associated with lower and slower disease progression and less severe internal organ involvement compared to diffuse cutaneous SSc [77]. Despite this, our cohort had statistically significantly lower NBSs than the general population in all domains, except in terms of general mental health and role limitations because of emotional problems, and were within the lowest NBSs among the other nine chronic diseases. This indicates that even patients predominantly affected by milder SSc phenotypes experience significant impairment in physical functioning and overall quality of life, meaning that a cohort with a larger diffuse cutaneous SSc representation might have yielded even worse HRQoL scores.

## CONCLUSIONS

Our comparison of HRQoL in patients with SSc to that of patients with leading causes of global disease burden highlighted the substantial impact of SSc, which ranked among the four diseases with the poorest physical functioning. Patients with SSc also reported the poorest mental health among the non-psychiatric chronic diseases, comparable to that reported by patients with RA. Patients with SSc reported some of the lowest NBSs across the eight health domains of the SF-36, with significantly worse general health perception than any other disease, worse bodily pain than the patients with any of the six leading causes of the global disease burden, and higher impairment than most of the patients with the other nine diseases in terms of vitality and mental health. Role limitations because of physical problems among patients with SSc were comparable to those of patients with stroke, RA, and LC, while limitations because of emotional problems were similar to those of individuals with RA and stroke. The physical functioning and mental health impairment in patients with SSc were similar to that experienced by stroke and RA patients – two diseases with the most impaired HRQoL. These results highlight the need for further research to explore the factors that affect QoL in patients with SSc and identify those that are modifiable, as well as tailor interventions and services to improve patients’ health and QoL.

## Additional material


Online Supplementary Document

## Data Availability

**Data availability:** Primary participant-level data were available only for the SSc cohort included in this study. These data are not publicly available due to ethical and confidentiality restrictions, but may be made available in deidentified form upon reasonable request to the corresponding author, subject to appropriate approvals. Data for all other disease groups were extracted from published literature, and the original sources are cited within the manuscript.
