# Supplementary material for: Systemic sclerosis markedly impairs health-related quality of life: a single-centre tertiary cohort study with literature-based comparison to leading causes of global disease burden
Source: J Glob Health. 2026 Oct 23;16:04228. doi: 10.7189/jogh.16.04228 (PMC13693295; doi:10.7189/jogh.16.04228)
Supplement: Supplementary file 1 [file jogh-16-04228-s001.pdf]

Supplement to: Alcacer-Pitarch B, Duarte-Fernandes R, Redmond A, Rocamora-Gisbert C, Hensor E, Del Galdo F, Buch MH, Keenan AM. Systemic sclerosis markedly impairs health-related quality of life: a single-centre tertiary cohort study with literature-based comparison to leading causes of global disease burden. J Glob Health. 2026;16:04228.

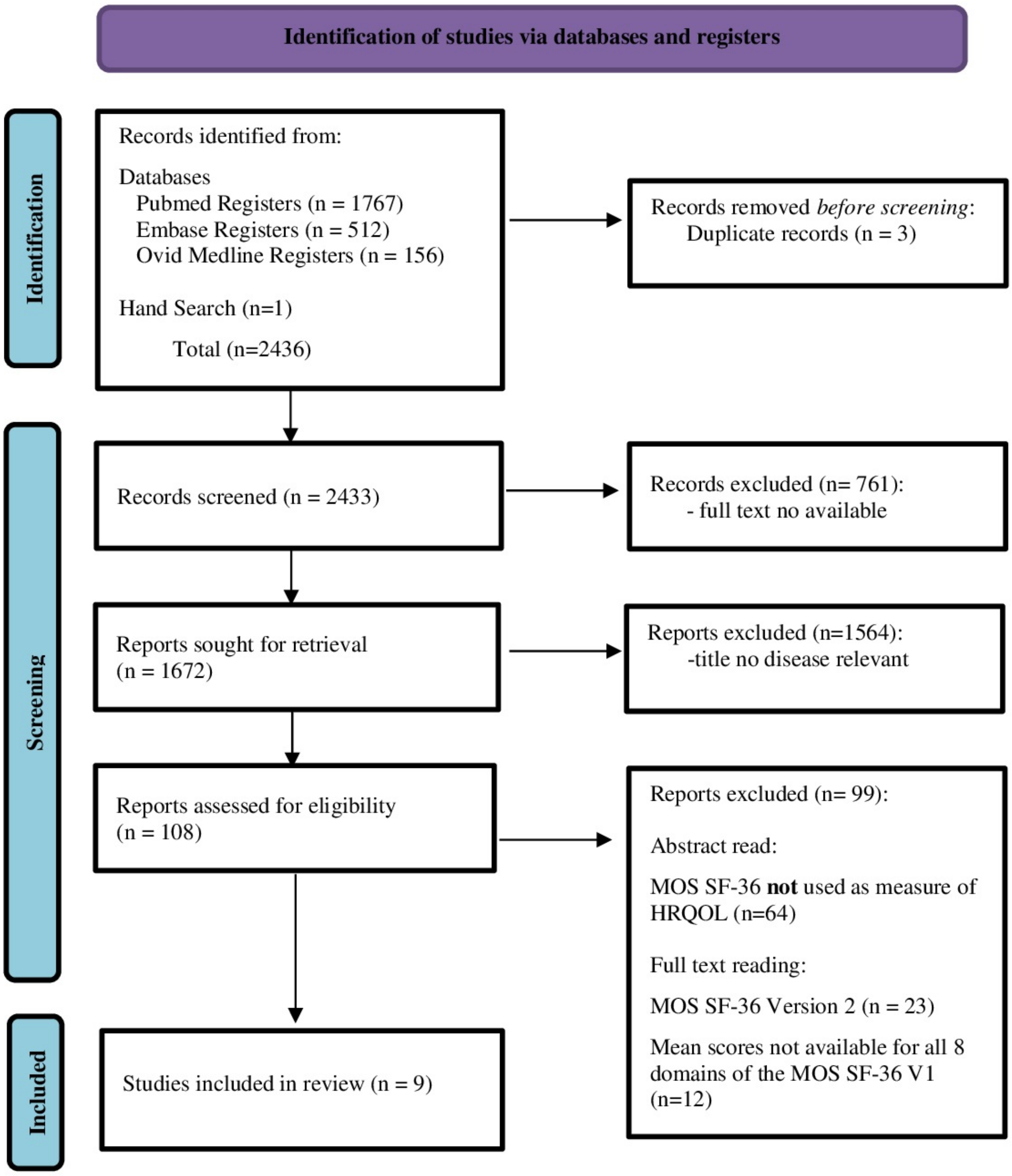

Figure S1. Literature search flowchart for selection of the included studies.

Text S1. Explanation of authorship change statement

The author CRG was added as a co-author after the submission of the manuscript due to her contributions in responding to the peer reviewers' comments. Specifically, CRG contributed in expanding the literature review within the introduction, especially in terms of how health-related quality is defined and evaluated. She also expanded on the study limitations by addressing reviewer concerns regarding how advances in care and treatment over time, differences between subgroups of scleroderma patients, and the capacity of the SF-36 questionnaire's mental health items to capture SSC-specific burden might have influenced the study results. To support the request for this change to the authorship byline, the authors have provided a signed authorship form and a revised authorship contribution statement, as well as manuscript versions and responses to peer reviewers' comments that reflect these contributions.
